# Techno-economic analysis of an integrated biorefinery to convert poplar into jet fuel, xylitol, and formic acid

**DOI:** 10.1186/s13068-022-02246-3

**Published:** 2022-12-20

**Authors:** Gabriel V. S. Seufitelli, Hisham El-Husseini, Danielle U. Pascoli, Renata Bura, Richard Gustafson

**Affiliations:** School of Environmental and Forest Sciences, University of Washington, Seattle, WA 98195 USA

**Keywords:** Poplar, Biorefinery, Techno-economic analysis, Sustainable aviation fuel, Jet fuel, Formic acid, Xylitol

## Abstract

**Background:**

The overall goal of the present study is to investigate the economics of an integrated biorefinery converting hybrid poplar into jet fuel, xylitol, and formic acid. The process employs a combination of integrated biological, thermochemical, and electrochemical conversion pathways to convert the carbohydrates in poplar into jet fuel, xylitol, and formic acid production. The C5-sugars are converted into xylitol via hydrogenation. The C6-sugars are converted into jet fuel via fermentation into ethanol, followed by dehydration, oligomerization, and hydrogenation into jet fuel. CO_2_ produced during fermentation is converted into formic acid via electrolysis, thus, avoiding emissions and improving the process’s overall carbon conversion.

**Results:**

Three different biorefinery scales are considered: small, intermediate, and large, assuming feedstock supplies of 150, 250, and 760 dry ktonne of poplar/year, respectively. For the intermediate-scale biorefinery, a minimum jet fuel selling price of $3.13/gallon was obtained at a discount rate of 15%. In a favorable scenario where the xylitol price is 25% higher than its current market value, a jet fuel selling price of $0.64/gallon was obtained. Co-locating the biorefinery with a power plant reduces the jet fuel selling price from $3.13 to $1.03 per gallon.

**Conclusion:**

A unique integrated biorefinery to produce jet fuel was successfully modeled. Analysis of the biorefinery scales shows that the minimum jet fuel selling price for profitability decreases with increasing biorefinery scale, and for all scales, the biorefinery presents favorable economics, leading to a minimum jet fuel selling price lower than the current price for sustainable aviation fuel (SAF). The amount of xylitol and formic produced in a large-scale facility corresponds to 43% and 25%, respectively, of the global market volume of these products. These volumes will saturate the markets, making them infeasible scenarios. In contrast, the small and intermediate-scale biorefineries have product volumes that would not saturate current markets, does not present a feedstock availability problem, and produce jet fuel at a favorable price given the current SAF policy support. It is shown that the price of co-products greatly influences the minimum selling price of jet fuel, and co-location can further reduce the price of jet fuel.

**Supplementary Information:**

The online version contains supplementary material available at 10.1186/s13068-022-02246-3.

## Introduction

According to the U.S. Energy Information Administration (EIA), in 2021, 79% of the energy consumption in the U.S. was from fossil fuel resources (crude oil, natural gas, and coal) [1]. In the same year, the combustion of fossil fuels in the U.S. accounted for 92% of the net human-induced CO_2_ emissions (4857 out of 5256 million metric tons of CO_2_ equivalent) [2]. The transportation sector contributes to approximately 37% of these emissions, with 97% from petroleum products (based on 2021 estimates [3]). One promising approach to reducing emissions in the transportation sector is to create process pathways that produce fuels from renewable biomass feedstocks that sequester carbon during growth. Great efforts have been made to decarbonize the road transportation sector, introducing low-carbon fuels, such as ethanol and biodiesel, and electrifying the sector. However, to date, only a few low-carbon fuel alternatives have been implemented for the aviation sector. Currently, the U.S. is mobilizing academia, industry, and government to develop sustainable, efficient, and economically feasible processes to produce sustainable aviation fuel from renewable resources. Recently, the Airline for America has announced the ambitious goal to achieve a production of 3 billion gallons of cost-competitive sustainable aviation fuel (SAF) by 2030, which corresponds to 15–20% of the current annual jet fuel production in the U.S. [4]. This plan would positively impact the aviation sector and help reduce emissions and dependence on fossil fuels.

In 2016, the U.S. Department of Energy (DOE), in partnership with the Oak Ridge National Laboratory, released the *2016 Billion-Ton Report* [5]. According to the report, the U.S. can supply 1 billion tons of renewable resources (agricultural and forestry residues, energy crops, algal, and waste) as feedstock for biofuels, biochemicals, and biomaterials by 2040. Although the feedstock supply available in the U.S. is sufficient to produce 3 billion gallons of SAF by 2030, achieving a cost-competitive jet fuel selling price starting from renewable resources is challenging. Unlike fossil resources (petroleum, coal, and natural gas), biomass resources (agricultural and forestry residues, energy crops, algal, and waste) are heterogeneous and require considerable processing to produce infrastructure compatible with hydrocarbon fuels. Conversion of renewable resources into SAF requires several processing steps, including fractionation, conversion, recovery, and purification, making the process expensive. Additionally, these processes are unable to convert all the carbon available in the feedstocks, and the remaining carbon becomes process waste, an emission, or is used for electricity and heat.

Different pathways have been proposed to convert renewable feedstocks into SAF, including Alcohol-to-Jet (ATJ), Fischer–Tropsch from syngas, hydroprocessing of fats and fatty acids, and hydrogenolysis of lipids [6, 7]. While the hydroprocessing of fats is currently the main technology used to produce SAF in the U.S., the limited capacity of fat waste is expected to hinder the further scale-up of this technology. The ATJ process is considered a viable alternative to the capacity issue due to the large availability of ethanol [8, 9] and the growing number of proposed ATJ projects to be developed in the United States.

Alcohol-to-jet processes using cellulosic feedstocks are especially promising due to the low life cycle carbon emissions when producing cellulosic ethanol [10]. The economics of producing SAF from cellulosic ethanol, however, are challenging. Previous research from the National Renewable Energy Laboratory (NREL) concluded ethanol from corn stover would have a minimum fuel selling price (MFSP) of $2.15/gallon [11], assuming a modest discount rate of 10%. Achieving the target SAF price of $2.50/gallon—the current target set by the Department of Energy (DOE) [7]—from lignocellulosic ethanol at $2.15/gallon is unrealistic since there will be yield losses and substantial operating and capital costs associated with converting ethanol to jet fuel. Innovative strategies that lower jet fuel production costs are necessary to make the ATJ process economically feasible with lignocellulosic biomass.

There is a consensus that one solution to economically feasible biofuels involves biorefinery integration to produce a diverse portfolio of products [12], especially co-products that have substantially higher value than jet fuel. This biorefinery will have superior economics because it produces higher value products and enables more complete use of the biomass resource for chemicals, fuel, and energy. From a technical and operating standpoint, however, achieving an efficient integrated biorefinery process design is challenging because of interactions between individual process units. Further, the lack of infrastructure and issues with market development penetration for bioproducts create a barrier to implementing integrated biorefineries [12, 13]. The present work presents a holistic approach to this problem by converting lignocellulosic biomass into jet fuel and co-products with well-established markets, resulting in favorable overall process economics.

In 2019, Rosales-Calderon and Arantes [14] published an excellent review on chemicals and materials that are produced at commercial scales and that could be immediately co-produced with lignocellulosic ethanol. These products include polyols, alcohols, furfurals, organic acids, and alditols. For example, polyols, such as 1,2-butanediol and 1,4-butanediol, have an average selling price of $2,900/tonne [15], and 2,3-butanediol—a high-value polyol [16]—has a market volume of 32 million tonnes/year [17, 18]. Alditols, such as sorbitol and xylitol, have selling prices of $720/tonne [19] and $4,400/tonne [19] (price estimate from 2015—current price for xylitol may reach values as high as $6,500/tonne), respectively, and attractive markets for use in food and pharmaceutical products [14]. Although organic acids are not high-value compared to polyols and alditols, they have strong markets with large volumes—for example, acetic acid has a market volume of 8.3 million tonnes/year [20]. Co-producing jet fuel and some of these products (i.e*.*, organic acids, polyols, and alditols) could create a viable process pathway for cost-competitive jet fuel.

This paper presents the techno-economic analysis (TEA) of an integrated biorefinery to convert poplar wood into xylitol, formic acid, and jet fuel. A primary biorefinery design objective is process integration that maximizes biomass utilization and minimizes CO_2_ emissions. The technical aspects of the processes to convert the biomass into jet fuel, xylitol, and formic acid are thoroughly discussed. Several TEAs have been published addressing the conversion of renewable feedstocks into jet fuel [21–28] and bioproducts [27, 29], but only a few attempts [15, 30] have been made to design integrated processes to co-produce jet fuel and high-value products from lignocellulosic biomass. As discussed in the present publication, this approach substantially lowers the jet fuel selling price and can establish a feasible process design for SAF to achieve the current DOE target price of $2.50/gallon by 2030.

## Results and discussion

### Biorefinery scale

Detailed supply curves for poplar biomass for a Lewis County (WA) biorefinery have been developed in a recent study [31]. They showed that up to 760 ktonne/year of poplar wood, primarily grown on land designated as pastureland, would be available for the factory. For our analysis, we assumed an intermediately sized biorefinery that uses 250 dry ktonne/year (685 dry tonne/day) at an average plant-gate biomass cost of $77 per dry tonne. This constitutes our base case. Then we also assessed a small-scale biorefinery operating at a biomass feed rate of 150 dry ktonne/year (411 dry tonne/day) with an average plant-gate cost of $65 per dry tonne and a large-scale biorefinery operating at the maximum biomass availability in Southwest Washington, 760 dry ktonne/year (2082 dry tonne/day) with an average plant-gate cost of $85 per dry tonne.

Table 1 shows the jet fuel, formic acid, and xylitol production as a function of biorefinery feedstock capacity for the 3 biorefinery scales considered in this study (small-scale, intermediate-scale, and large-scale). As expected, the results show that the product capacities are proportional to biorefinery feedstock capacity. For the 3 scales (small, intermediate, and large), the percent conversions of carbon in the biomass into jet fuel, xylitol, and formic acid are 22, 14, and 14 C%, respectively. Lignin, which corresponds to a substantial fraction of the carbon present in the biomass, 37 C%, is used for heat and electricity production.

**Table 1:** Jet fuel, formic acid, and xylitol capacities as a function of biorefinery feedstock capacity

Biorefinery feedstock capacity (dry ktonne/year)	Jet fuel (MMgal/year)	Formic acid (ktonne/year)	Xylitol (ktonne/year)
150 (small-scale)	7	38	16
250 (intermediate-scale)	11	64	27
760 (large-scale)	35	193	82

Jet fuel is a product with a huge market—the global market volume for jet fuel is estimated at 106 billion gallons/year [7], much larger than the production obtained for the 3 cases. Even for a large-scale biorefinery, the jet fuel market could easily accommodate the fuel produced in the biorefinery. The situation for formic acid and especially xylitol is different. Although formic acid is a product with a strong market and many applications, the formic acid capacity for the large-scale biorefinery corresponds to 25% of its global market volume, estimated at 762 ktonne/year [32]. The xylitol market is still under development, with a global market volume estimated at 190 ktonne/year [33]. The xylitol production volume in a large-scale biorefinery would, therefore, correspond to 43% of the global xylitol market volume, creating a huge barrier to market entry. It is important to note that large xylitol and formic acid volumes from the biorefinery may create an opportunity to lower the xylitol and formic acid selling prices, currently estimated at $4,200/tonne [14, 33] and $1,000–1,200/tonne (current price in the U.S.—formic acid prices in China are estimated at $400–600/tonne), respectively, creating an opportunity to expand the market volume for these products. Large market volumes could increase the adoption of xylitol in foodstuff and hygiene products and create a pathway for domestic formic acid production from renewable resources as an alternative to petroleum-based formic acid. From a market volume perspective, the large-scale biorefinery appears unrealistic, and there would be considerable challenges to sourcing 760 ktonnes of appropriate biomass per year for a single biorefinery.

Table 2 shows the total installed equipment cost of the primary biorefinery areas as a function of biorefinery feedstock capacity. The relative contribution of the individual biorefinery areas to the total installed equipment cost is only moderately dependent on feedstock capacity. The areas associated with heat and electricity generation (A900) and wastewater treatment (A1000) account for 44% of the total installed equipment cost. Thus, co-locating the biorefinery with a power plant can reduce the total installed equipment cost of the biorefinery by as much as 28%. The cost to fractionate biomass into C_5_ and C_6_ sugars and lignin, including biomass fractionation (A100) and saccharification (A200), accounts for approximately 22% of the total installed equipment cost. This analysis shows that the biggest capital cost drivers are heat/electricity production, wastewater treatment, and biomass fractionation (65% of the total fixed capital). Jet fuel production (A300, A400, A500, and A600) contributes to 13–16% of the total fixed capital, with ethanol production (A300) accounting for approximately 39–54% of the installed equipment expense, among the process steps to convert the C_6_ sugars (primarily glucose) into jet fuel. Xylitol and formic acid production areas—A700 and A800, respectively—each account for approximately 10% of the total installed equipment cost.

**Table 2:** Installed equipment cost (in million $) for the individual areas of the biorefinery and total capital investment as a function of biorefinery feedstock capacity

Biorefinery feedstock capacity (dry ktonne/year)	Fixed capital (million $)		
	150 (Small-scale)	250 (Intermediate-scale)	760 (Large-scale)
Biomass fractionation (A100)	33	39	59
Saccharification (A200)	16	25	65
Fermentation (A300)	20	22	30
Alcohol dehydration (A400)	3	5	10
Oligomerization (A500)	4	6	14
Hydrogenation (A600)	10	13	23
Xylitol production (A700)	23	32	65
Formic acid production (A800)	18	28	74
Boiler/turbogenerator (A900)	63	86	168
Wastewater treatment (A1000)	35	47	94
Total installed equipment cost	225	303	602
Total capital investment	418	559	1109

**Fig. 1:**
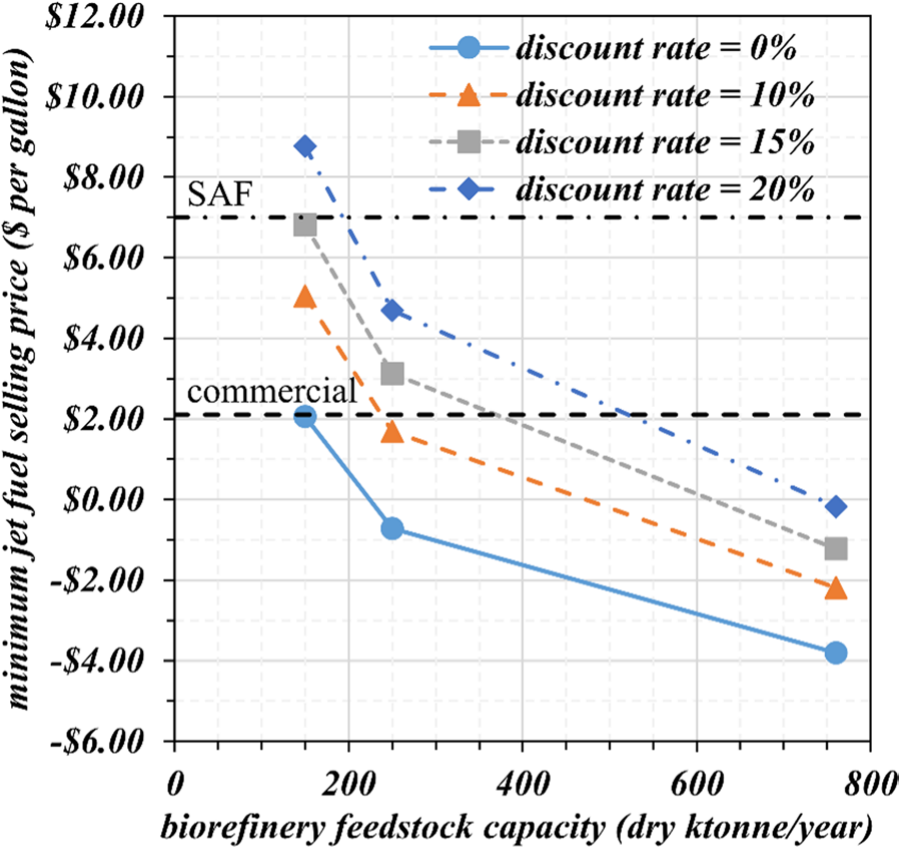
Minimum jet fuel selling price in $ per gallon as a function of biorefinery feedstock capacity for the 3 scales assumed in the present study (small, intermediate, and large) and discount rates of 0 (break-even cost), 10, 15, and 20%. Baseline prices of $1,000 and $4,200 per tonne were assumed for formic acid and xylitol, respectively

Figure 1 shows the minimum jet fuel selling price as a function of biorefinery feedstock capacity at discount rates of 0 (break-even cost), 10, 15, and 20%, assuming fixed formic acid and xylitol selling prices of $1,000 and $4,200 per tonne (current market selling prices), respectively. The result shows that jet fuel production at cost (discount rate of 0%) leads to a minimum jet fuel selling price below the current commercial price for all biorefinery capacities. The small-scale biorefinery is profitable at discount rates of 10 and 15%, leading to a minimum jet fuel selling price lower than that of SAF, $7.00/gallon [34]. At a discount rate of 20%, the small-scale biorefinery is not feasible since the jet fuel selling price is higher than the current SAF price. The intermediate-scale biorefinery shows favorable jet fuel selling prices. At a low discount rate of 10%, the minimum jet fuel selling price is lower than the price of commercial jet fuel. At a more realistic discount rate of 15%, the minimum jet fuel selling price is $3.13/gallon, which gives a $3.87/gallon margin relative to the policy-supported SAF price. Even at the highest discount rate assumed in this study, 20%, the jet fuel selling price remains below the current supported SAF price for an intermediate-scale biorefinery. In an optimistic scenario of maximum biomass availability (large-scale biorefinery scenario), the minimum jet fuel selling price is lower than the current jet fuel selling price at all discount rates.

The large-scale biorefinery operating at maximum biomass capacity exhibits an outstanding jet fuel selling price, but at this capacity, the co-products, xylitol and formic acid, will have challenging market entry issues, as previously discussed. Further, the total capital investment for building the large biorefinery exceeds $1.1 billion, which imposes a barrier to the feasible implementation of this enterprise and is a high-risk investment at the current stage of this technology. It appears from our analysis that the small and intermediate-scale biorefineries are the most viable from a feedstock, an economic, and a market volume perspective. We focus on the intermediate-scale biorefinery (base case) for the following discussions because it presents better economics than the small-scale factory.

### Process utilities

**Fig. 2:**
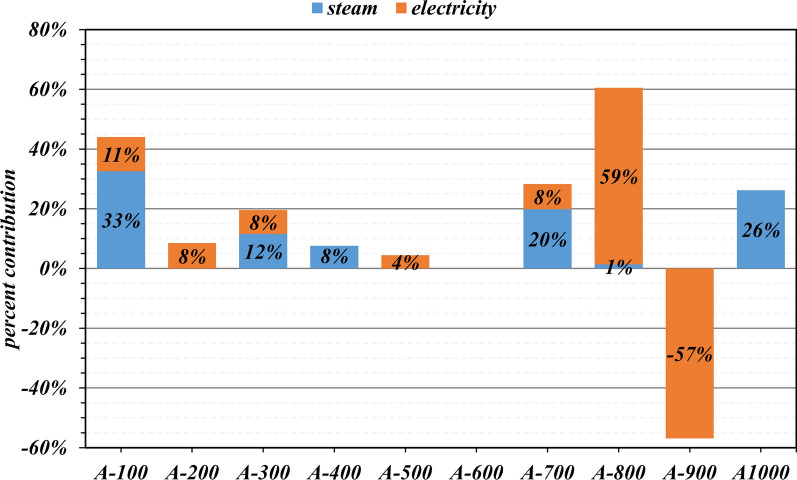
Relative utility usage (steam and electricity) of individual biorefinery areas for the intermediate-scale biorefinery (base-case scenario). A100—biomass fractionation, A200—saccharification, A300—ethanol production, A400—alcohol dehydration, A500—oligomerization, A600—hydrogenation, A700—xylitol production, A800—formic acid production, A900—boiler and turbogenerator, and A1000—wastewater treatment

Figure 2 presents the relative demand for steam and electricity in individual biorefinery areas for the intermediate-scale biorefinery. The total utility requirements for the biorefinery are presented in Table 3. According to the results shown in Fig. 2, biomass fractionation (A100) and wastewater treatment (A1000) account for most of the steam requirements of the biorefinery—33 and 26%, respectively. Xylitol production (A700) is also a heat-intensive process due to the multiple-effect evaporators used to remove furfurals, organic acids, and other impurities from the C_5_ sugar stream, accounting for 20% of the total steam usage. Steam is also necessary for the reboilers of the distillation columns used to separate the ethanol from water in A300, accounting for 12% of the total steam requirement of the biorefinery.

**Table 3:** Summary of total utility requirements for the intermediate-scale biorefinery (base case)

	Steam (tonne/hr)	Electricity (kW)
Total requirement	305	45000

The conversion of CO_2_ and water into formic acid in A800 accounts for 59% of the total electricity requirement in the modeled biorefinery. Areas A100, A200, A300, and A700, require approximately the same amount of electricity, contributing to 8–11% of the total electricity required for pumps and compressors. In the present design, the steam required for all areas of the biorefinery is produced in the biorefinery by burning make-up nature gas, lignin produced from the biomass, and syrup from wastewater treatment, contributing to 60, 23, and 17% of the total energy for steam production, respectively. The superheated steam is used in the turbogenerator to produce 26,000 kW of electricity, which is sufficient to power all the areas of the biorefinery, except for A800, due to the great amount of electricity required to run the electrochemical reactor; thus, an additional 19,000 kW of electricity is supplied from the grid.

### Sensitivity analysis and co-location

The effect of independent process variables (one at a time) on the minimum jet fuel selling price is analyzed. Different scenarios are explored in this section, including changes in market-related and technical-based parameters. Changes in biomass, electricity, enzyme, and natural gas cost (these 4 combined correspond to 91% of the total variable operating costs) and xylitol and formic acid selling prices are considered to assess the impact of market-related parameters on the selling price of jet fuel. For technical-based parameters, changes in conversion, yields, and product recovery are considered. The results presented in this section are compared to the minimum jet fuel selling price (calculated at a discount rate of 15%) for the intermediate-scale biorefinery (base-case scenario, assuming a biorefinery operating at a biomass availability of 250 dry ktonne/year), $3.13/gallon. A disturbance of ± 25% for the independent variables analyzed is assumed in the sensitivity analysis.

We also investigated a scenario where the biorefinery is co-located with a power plant. In this scenario, the biorefinery does not produce steam and electricity; these two inputs are supplied from a power plant. Prices of $0.04/kWh and $7/tonne were assumed for electricity and steam (at 280 °C and 1310 kPa), respectively, for co-location with a power plant. Also, the power plant would burn the lignin stream originally used for heat and electricity generation in the base-case scenario.

**Fig. 3:**
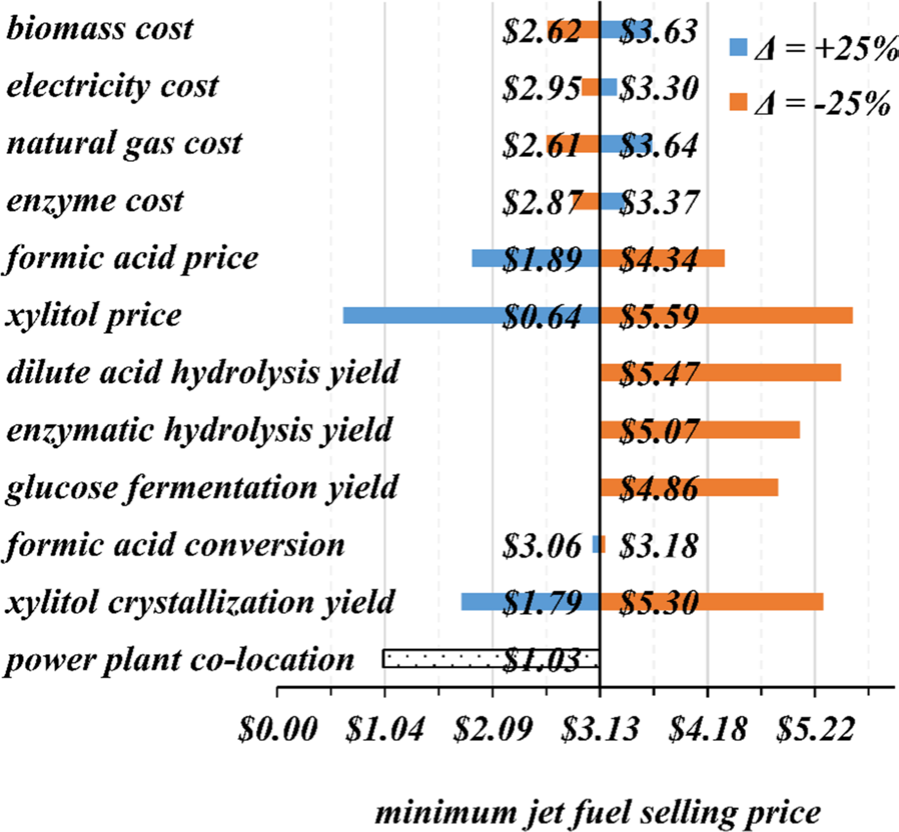
Change in minimum jet fuel selling price for a disturbance of ± 25% in biomass, electricity, enzyme, and natural gas costs, formic acid and xylitol selling prices, yields of dilute acid hydrolysis, enzymatic hydrolysis, glucose fermentation, and xylitol crystallization, and formic acid conversion relative to the jet fuel baseline price of $3.13/gallon for the intermediate-scale biorefinery. The graph also shows the change in jet fuel selling price with co-location with a power plant

Figure 3 shows that the minimum jet fuel selling price is highly sensitive to changes in the co-product prices. A favorable scenario where xylitol is sold at approximately $5,250/tonne (25% higher than its current market price, $4,200/tonne) would lead to a jet fuel price much lower than that of commercial jet fuel. Note that the jet fuel selling price remains below the current SAF price, $7.00/gallon, for all scenarios considered. Based on our analysis, co-products are crucial to reducing the jet fuel selling price and making SAF production feasible from an economic perspective.

Biomass and natural gas are the primary resources that contribute to the jet fuel price, as shown in Fig. 3. A realistic lower-cost biomass scenario involves co-utilizing hybrid poplar for wastewater treatment and as feedstock for the biorefinery [31]. This approach could lower the feedstock price by as much as 15%, thus reducing the overall plant-gate cost of the biomass.

Figure 3 also shows that a decrease of 25% in the yields of dilute acid hydrolysis, enzymatic hydrolysis, glucose fermentation, and xylitol crystallization (one at a time) lead to significant increases of $5.47, $5.07, $4.86, and $5.30 per gallon of jet fuel, respectively. An exception is an approximately negligible change in jet fuel selling price with a disturbance of ± 25% in formic acid conversion in the electrochemical reactor. Our base-case design assumes that the yields of the processes investigated in the sensitivity analysis are maximized based on previous designs reported in the literature and previous work done at the University of Washington. The analysis of the effect of technical-based parameters on the minimum jet fuel selling price shows that if optimum yields are not achieved, it could drastically impact the economics of the process. Nevertheless, even if optimum yields are not achieved, the minimum jet fuel selling price would still be lower than the current price of subsidized SAF.

One important consideration for the biorefinery designed in the present study is the co-location with existing facilities that could provide the utilities and resources to run the process. Co-location with a power plant could provide the necessary steam and electricity to run the processes and lower the jet fuel selling price. As shown in Fig. 3, this scenario substantially reduces jet fuel selling price (70% reduction), making the process more viable from both a technical and economic perspective since a boiler and a turbogenerator would not be necessary. It is important to note, however, that depending on the source of fuel used in the power plant, i.e., biogenic versus non-biogenic, the economic benefit of co-locating the biorefinery with the power plant would come at the expense of burning fossil fuels, thus leading to a SAF that may not meet greenhouse gas emission standards.

While not specifically investigated in this study, it has been found that co-locating the biorefinery with existing crude oil refineries would decrease its capital investment by providing the necessary hydrotreating and hydrocarbon fractionation units (hydrotreating equipment such as reactors are available in crude oil refining facilities). According to a recent publication, this approach would reduce the jet fuel selling price by 3–23% [35].

## Methods

### Feedstock

The feedstock is one of the primary drivers of biorefinery’s operating costs [11]. Hybrid poplar is abundant in the Northwest region [5] and has been considered one of the main energy crops for biofuels and biochemicals in the U.S [5, 36] due to its excellent characteristics—high sugar and low ash contents. Also, poplar requires low fertilizer input, can re-sprout after multiple harvests, and has a high growth rate and large biomass accumulation [21, 36, 37]. While most poplar production is from forestry and farm lands [37], marginal lands have been considered a good alternative to growing energy crops for biofuels and biochemical [38].

Table 4 presents the composition of the hybrid poplar feedstock considered in this publication based on previous works conducted at the University of Washington [37].

**Table 4:** Chemical composition of hybrid poplar chips assumed in the model [37, 39]

	Chemical composition (%)									
	Cellulose	Xylan	Mannan	Galactan	Arabinan	Total sugar	Total phenolics	Acetic acid	Ash	Extractives
Hybrid poplar chips	46.5	13.1	2.6	0.4	0.4	63.0	25.3	1.2	0.7	5.2

### Feedstock fractionation (A100 and A200)

**Fig. 4:**
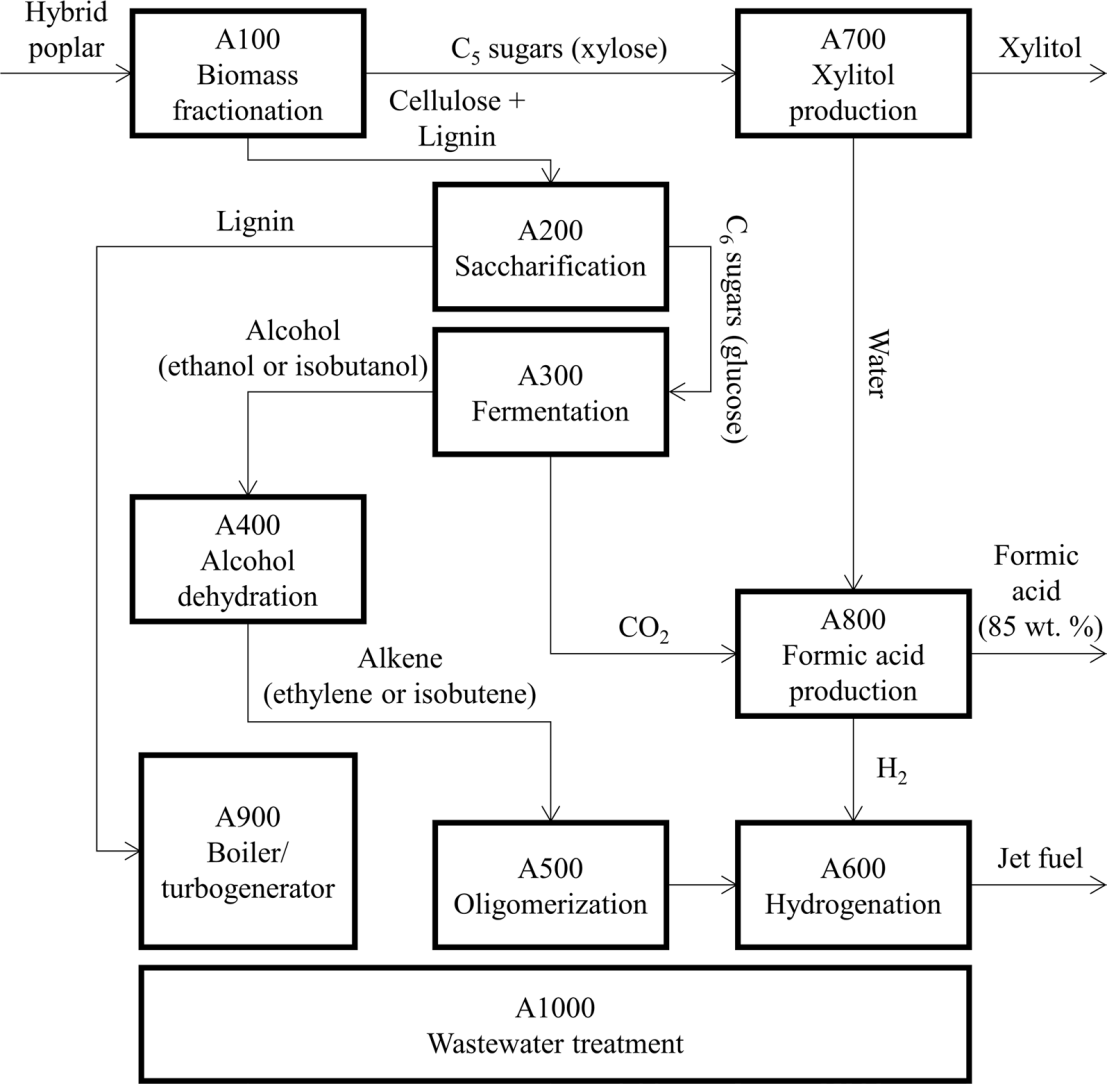
Process flow diagram of the integrated biorefinery showing the primary process areas and a simplified schematic of the process streams

The process flow diagram of the envisioned biorefinery is depicted in Fig. 4. Feedstock fractionation includes areas 100 (Fig. 4—A100 Biomass Fractionation) and 200 (Fig. 4—A200 Saccharification). Biomass chips are fractionated into cellulose, hemicellulose, and lignin by dilute acid hydrolysis at 195 °C and 13 bar with formic acid [40] in A100. The choice of an organic acid during acid hydrolysis is to avoid inorganics in downstream processes (it is well-known that inorganic compounds are troublesome in catalytic and biological conversion processes) and because formic acid is one of the main products of the biorefinery—approximately 12% of the formic acid produced in the facility is used for biomass pretreatment. The liquid (containing hydrolyzed sugars—mostly xylose) and solid (containing cellulose, lignin, and ash) phases are separated by washing with process water. The washer unit was chosen in this study to avoid costly solid–liquid separation [11] and because it is a widely implemented unit operation in the pulp and paper industry. Also, the washer allows for high xylose recovery. We selected a washer with a Norden number of 18 operating with a dilution factor of 0.6. This configuration provided a washing yield of 98%. This design was successfully modeled using WinGems and implemented in Aspen as a separation block (Sep block in Aspen). The liquid stream from A100 goes to area 700 (Fig. 4—A700 xylitol production), and the solid stream goes to A200.

The cellulose in the washed solids is hydrolyzed into glucose at 48 °C and a cellulase loading of 20 mg/g of cellulose [11]. The solids in the hydrolysate (mostly lignin and ash) are separated using a filter press and are sent to area 900 (Fig. 4—A900 boiler/turbogenerator) to produce steam and electricity, and the hydrolysate goes to area 300 (Fig. 4—A300 fermentation) for fermentation of the C_6_ sugars (primarily glucose). The present design assumes that the cellulase is produced on-site, consistent with modeling approaches in previous TEA studies [11]. Table 5 presents the chemical reactions and conversions assumed for the acid hydrolysis of biomass in A100 and enzymatic hydrolysis in A200. Additional file 1: Figures S1 and S2 show the detailed process flow diagrams of A100 and A200, respectively.

**Table 5:** Dilute acid hydrolysis and enzymatic hydrolysis reactions and conversions

Reaction	Reactant	% Converted into product
Dilute acid hydrolysis (A100)		
(Cellulose)_n_ + n H_2_O → n glucose	Cellulose	5.0%
(Xylan)_n_ + n H_2_O → n xylose	Xylan	90.0%
(Xylan)_n_ → n furfural + n H_2_O	Xylan	5.0%
Acetate → acetic acid	Acetate	100.0%
(Lignin)_n_ → n soluble lignin	Lignin	5.0%
Enzymatic hydrolysis (A200)		
(Cellulose)_n_ + n H_2_O → n glucose	Cellulose	90.0%

### Alcohol synthesis (A300)

In A300, the hydrolyzed sugars from A200 are fermented into ethanol. Fermentation of glucose into ethanol is a well-developed process and can employ bacteria (e.g*.*, *Zymomonas mobilis*) [11] or yeast (e.g*.*, *Saccharomyces cerevisiae*) [41]. From a technical standpoint, chemical processes employing bacteria or engineered microorganisms are higher risk due to the high chance of contamination [42]. Therefore, the present design employs the yeast *Saccharomyces cerevisiae* for fermenting glucose into ethanol due to the yeast’s high resistance to the product (ethanol) and the high fermentation yields achieved with this organism. Also, the concentration of C_5_ sugars at the inlet stream of the fermentation process in A200 is small, which favors the use of *Saccharomyces cerevisiae*. It is important to note that commercial processes employing *Saccharomyces cerevisiae* are well established. The fermentation broth exits the fermenter with an ethanol concentration of 3 wt. % and is sent to the first distillation column (denoted beer column) for ethanol recovery. The design for the beer column is similar to the one used in the NREL’s report from 2011 [11]. The concentrated ethanol stream from the beer column (with an ethanol concentration of approximately 44 wt. %) is sent to a second distillation column, where ethanol is further concentrated to 92 wt. %. The outlet ethanol stream is sent to area 400 (Fig. 4—A400 alcohol dehydration) for dehydration into ethylene. Note that the concentration of inorganics and water after alcohol distillation in A300 is low or negligible, thus avoiding additional unit operations to clean the ethanol stream, such as molecular sieve packages. The CO_2_ produced during glucose fermentation is easily recovered in the overhead of the beer column, and it is sent to area 800 (Fig. 4—A800 formic acid production), where it is converted into formic acid by reacting it with water in an electrochemical reactor. The bottom streams of the beer column and the second distillation column (mostly water) are sent to area 1000 (Fig. 4—A1000 wastewater treatment) for wastewater treatment. Additional file 1: Figure S3 shows the process flow diagram of A300 for the ethanol production area.

### Alcohol-to-Jet (A400, A500, A600)

The Alcohol-to-Jet (ATJ) process involves the conversion of alcohols into hydrocarbon molecules suitable for jet fuel by 1) alcohol dehydration into light alkene gases in A400, 2) light alkene oligomerization into higher alkenes in area 500 (Fig. 4—A500 Oligomerization), and 3) hydrogenation of higher alkenes into alkanes in area 600 (Fig. 4—A600 hydrogenation). From a technical standpoint, oligomerization is the most challenging process among the ATJ process steps due to the low conversion of light alkenes into long-chain hydrocarbons and the high selectivity for lower molecular weight products (especially butene and hexene). Ethylene oligomerization especially imposes a challenge due to the higher degree of polymerization required to produce liquid hydrocarbons relative to oligomerization of higher alkenes, such as butane [43].

The 3 steps of the ATJ process (dehydration, oligomerization, and hydrogenation) employ heterogeneous catalysts. Alcohol dehydration usually employs acid catalysts, temperatures of 200–400 °C, and low or high pressure [44]. The present design employs a trifluoromethanesulfonic acid silica-based catalyst at 200 °C and 1 atm [45] for ethanol dehydration into ethylene, with an overall yield of 98% and excellent selectivity (> 99%) to production of ethylene as reported in the literature [45]. In the present design, a nickel-based heterogeneous catalyst (Ni-H-Beta, Ni-SBA15, or Ni-Siral) was used for ethylene oligomerization based on previous studies at the University of Washington [46–52]. Conversions and selectivities have been reported in [46–51] for ethylene oligomerization. The design of the unit operation of hydrogenation adopted in the present study was the same used in [43]. In our process, the concentration of water and inorganics at the inlet of A400 is small (below 5 wt. %), which is assumed small enough to avoid reducing catalyst performance during ethanol dehydration into ethylene. Additional file 1: Figure S4 shows the process flow diagrams for A400, A500, and A600.

### Xylitol production (A700)

Hydrogenation is employed to convert sugars into alditols [53]. Because the production of xylitol as target alditol requires a xylose-rich stream, the primary challenges in producing pure xylitol from mixed sugar streams are the necessary separation and recovery steps to isolate the target sugar before hydrogenation [53]. Usually, the hydrolyzed sugar stream from biomass pretreatment (C_5_-rich stream from A100) contains a mixture of xylose, glucose, arabinose, galactose, and mannose. Because the hybrid poplar used in the present design contains mostly glucose and xylose (Table 4), the amount of arabinose, mannose, and galactose in the C_5_-rich stream from A100 is small. Still, hydrogenation of this process stream without some additional processing will lead to a complex mixture of alditols, primarily composed of xylitol and sorbitol.

**Fig. 5:**
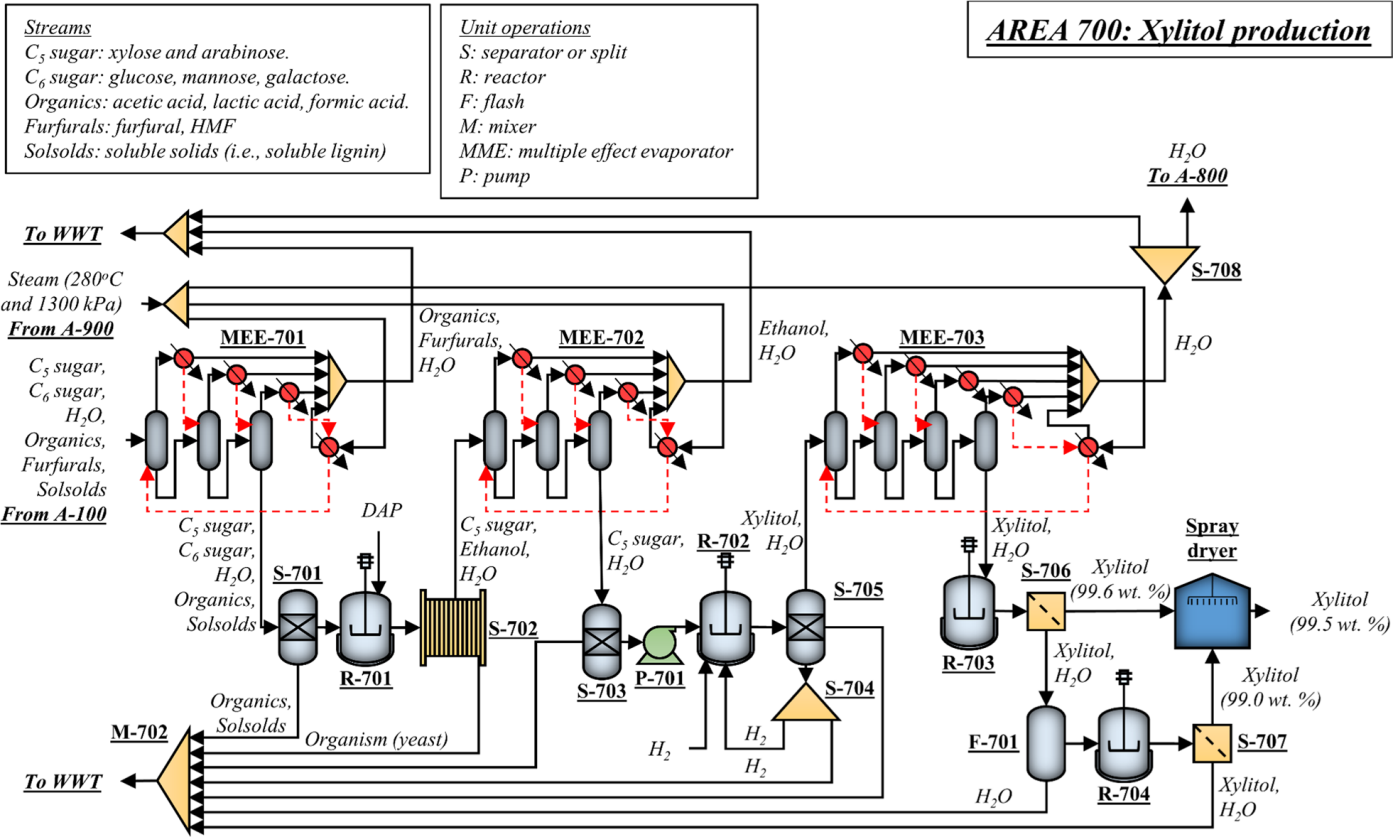
Process flow diagram of the xylitol production process designed in the present work

Crystallization of an alditol mixture is troublesome due to the slow growth and irregular shape of the target alditol crystals and slow filtration [53]. To avoid complications during crystallization, the maximum concentration of secondary alditols in the dissolved solids is kept below 15 wt. % [53]. The secondary sugars in the inlet of the hydrogenation reactor are usually separated by simulated moving beds (SMB) chromatography, which is not a well-developed commercial-scale unit operation. This study presents an innovative approach for converting liquid sugar streams into xylitol at high yields by combining biological and thermochemical conversion processes. The approach to clean the sugar stream and obtain a xylose-rich stream involves converting secondary sugars (mostly glucose in the designed biorefinery) into ethanol via fermentation. The fermentation process employs S*accharomyces cerevisiae* to avoid the consumption of xylose. Figure 5 shows the process flow diagram of the xylitol process designed in the present work.

The process starts with the liquid stream from A100. Initially, the furfurals (furfural and HFM) produced during biomass pretreatment in A100 are removed from the sugar stream by multiple-effect evaporators (Fig. 5—MEE-701). Part of the formic acid (used during pretreatment) and acetic acid (produced from the acetate groups in poplar) are also evaporated in this step. Then, the concentrate from the MEE is sent to an activated carbon column (Fig. 5—S-701), where part of the organic acids and dissolved lignin in the inlet stream are removed. The glucose in the sugar stream at the outlet of the activated carbon column is converted into ethanol via fermentation (Fig. 5—R-701). The fermentation is fast (~ 4–6 h) because of the low glucose concentration in the fermenter’s inlet stream, 10–15 g/L. The yeast is separated from the broth using a filter press (Fig. 5—S-702), and the ethanol is separated from the xylose-rich stream via MEE (Fig. 5—MEE-702). Another activated carbon column (Fig. 5—S-703) removes impurities from the sugar stream before hydrogenation. Note that the amount of ethanol produced in A700 is small, and recovering ethanol as a product is not economically feasible.

The liquid stream containing xylose (total dissolved solids = 20–25 wt. %; xylose concentration in dissolved solids = 99 wt. %) is pressurized to 125 bar (Fig. 5—P-701) and fed into the hydrogenation reactor (Fig. 5—R-702) [53]. Hydrogen is also fed into the reactor, and the unreacted hydrogen is recycled to maintain a hydrogen-to-xylose ratio 4 times the stoichiometric requirement. The effluent from the reactor is passed through an activated carbon column (Fig. 5—S-705) and then another MEE (Fig. 5—MEE-703) to remove impurities and water, respectively. The feed to the crystallizers (Fig. 5—R-703 and R-704, two crystallizers were used in the present design) contains 50–70 wt. % dissolved solids with a xylitol fraction in the dissolved solids of 98–99 wt. % [53]. The crystallizers operate at 8 °C. The xylitol crystals are recovered using centrifuges (Fig. 5—S-706 and S-707), and they are fed into a spray dryer (Fig. 5—spray dryer), where water is further removed. The crystals exit the dryer with a purity greater than 99 wt. %.

### Formic acid production (A800)

**Fig. 6:**
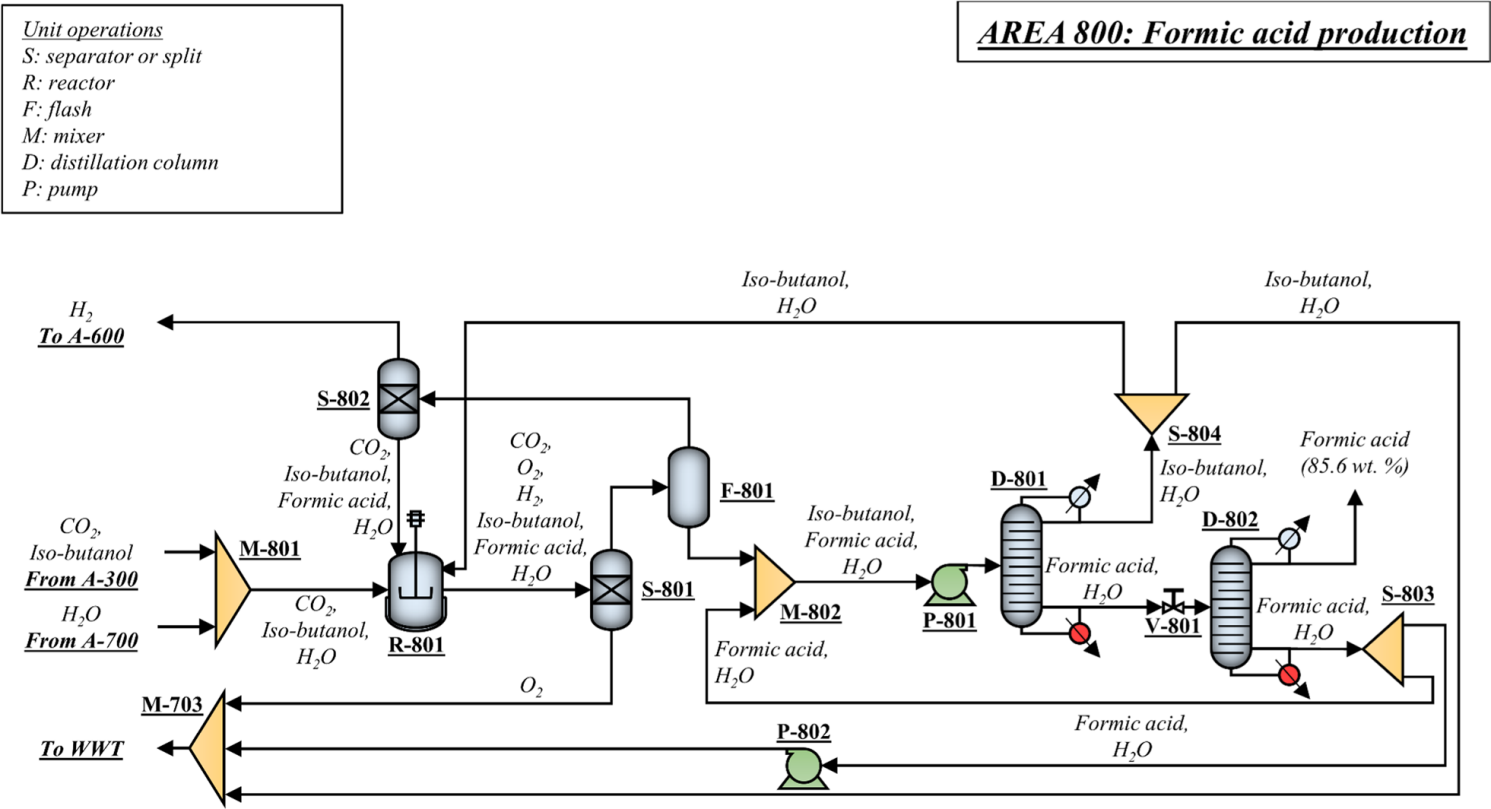
Process flow diagram of the formic acid production process designed in the present work

One of the main advantages of the integrated biorefinery proposed in the present study is the optimized use of carbon in the cellulose and hemicellulose biomass fractions for fuels and chemicals. The CO_2_ produced during fermentation in A300, which would become an emission, is sent to A800 for electrochemical conversion into formic acid. The evaporated water from A700 is used as a reactant in the electrocatalytic reactor—the amount of impurities in the water from A700 is negligible. Figure 6 shows the process flow diagram of the formic acid production process designed in the present work. The process adopted in the present design consists of an electrochemical cell (Fig. 6—R-801) designed by OCOChem [54, 55] that filters CO_2_ through a membrane and converts it into formic acid using electricity. A single-pass conversion of CO_2_ into formic acid of 10% is assumed. Based on personal communications with the CEO of OCOChem [54, 55], a faradaic efficiency of up to 90% can be achieved during the reduction of CO_2_ into formic acid with a cell voltage of 4.0 V and an average current density of approximately 125 mA/cm^2^, accounting to an energy requirement of approximately 4.3 kWh per kg of formic acid produced. For the electrochemical reactors, it is estimated a capital investment of $153/kW (assuming a current density of 300 mA/cm^2^) and $368/kW (assuming a current density of 125 mA/cm^2^). The process also produces H_2_ and O_2_ that can be separated using membranes (Fig. 6—S-801 and S-802). The conversion of water into H_2_ in the electrochemical reactor is 2%. The H_2_ produced in the electrochemical reactor is fed into the hydrogenation reactor in A600, but make-up H_2_ is still necessary to convert the remaining alkenes into alkanes. A price for hydrogen of $1368/tonne was used. Formic acid is separated from the water via pressure-swing distillation (Fig. 6—D-801 and D-802). Both columns contain 15 stages—the first one operates at 3 bar, and the second one operates at 0.4 bar. In the present design, formic acid at 85 wt. % is obtained.

### Boiler and turbogenerator (A900) and wastewater treatment (A1000)

The lignin-rich stream from A200 is sent to A900 and used as fuel for heat and electricity. Make-up natural gas is co-fed to produce the steam necessary to meet the total steam demand of the biorefinery. Electricity is co-produced and used to power most of the process areas of the biorefinery, except for the electrochemical reactor in A800. All the waste streams of the biorefinery are sent to A1000, where part of the water is evaporated using a 7-stage MEE [56]. The concentrate (syrup) is combusted in the boiler. Part of the treated water stream is used in A900 to produce steam. According to the present design, a small wastewater treatment plant is necessary to treat the remaining water stream not fed into the multiple-effect evaporator. We used NREL’s design from 2002 for wastewater treatment employing a combination of anaerobic and aerobic digestion [57]. The total capital investment for the additional water treatment plant is a small fraction of the total capital cost of A1000. Figure S5 shows the process flow diagram for A900.

### Techno-economic analysis

#### Cost-year indices

The Chemical Engineering Plant Cost Index (CEPCI) for the base year of 2019 was used for this analysis [58]. Equipment and operating costs were obtained from the literature and corrected to 2019$ using the CEPCI according to the following equation:

1 $$cost\left(2019\mathrm{\$}\right)=cost\left(base\$\right)\times \left(\frac{CE{PCI}_{2019}}{CE{PCI}_{base}}\right).$$

In Eq. 1, *cost(2019$)* is the updated equipment or operating cost (based on 2019$), *cost(base$)* is the cost at a given base year, and *CEPCI*_*2019*_ and *CEPCI*_*base*_ are the cost indexes for 2019, 619.2 [58], and the base year, respectively.

#### Equipment cost

Equipment costs were obtained from the literature [11, 57, 59, 60] and used to estimate total equipment costs for the individual unit operations and the total capital investment of the biorefinery. The installed equipment cost is based on the factored value of the equipment purchase cost, according to the following equation:

2 $$Installed cost=\left(purchased \, equipment\, cost\right)\times\, \left(multiplier\right).$$

Equation 2 does not include installation foundation, piping, and wiring costs [11]. These costs are factored in as a fraction of the total installed cost of Inside Battery Limit (ISBL).

Equation 3 was used to calculate the scaled cost of equipment relative to its base cost and size:

3 $$new cost=\left(base\, cost\right)\times {\left(\frac{New\, size}{Base\, size}\right)}^{n}.$$

In Eq. 3, *n* is a positive fraction and depends on the characteristics of the equipment (capacity, heat duty, or flow rate) [11]. Installed cost multipliers (Eq. 2) and characteristic scaling exponents (Eq. 3) were obtained from the literature [11, 57, 59, 60] and are listed in Table 6.

**Table 6:** Installation factor and characteristic exponents used in Eqs. 2 and 3 to calculate the installed and scaled costs of the individual unit operations

Equipment	Installation factors	Exponent
Mixer	1.00	0.50
Tank (flash, chemical addition, crystallizer)	2.00	0.70
Main fermenter	1.50	1.00
Seed fermenter	1.80–2.00	0.70
Heater	2.20	0.70
Boiler	1.80	0.60
Turbine	1.80	0.60
Washer	1.00	1.00
Filter press	2.30	0.80
Decanter	2.00	0.70
Multiple-effect evaporator	2.20	0.60
Spray dryer	1.00	0.70
Membrane	1.80	0.70
Centrifuge	1.00	0.60
Activated carbon filter	1.80	0.70
Hydrotreating facility	2.00	0.50
Column		
Beer	2.40	0.80
Hydrocarbon separation column	2.47	0.68
Condenser package	2.47	0.44
Reboiler package	2.47	0.68
Reactor		
Pretreatment	1.50	0.60
Electrocatalytic	1.00	1.00
Dehydration and oligomerization	2.47	0.65
Pump		
Water	3.10	0.80
Saccharification transfer	2.30	0.80
Crude hydrocarbons	2.47	0.79

Each area’s total installed equipment costs account for part of the total direct costs (TDC). Other TDCs include site development, warehouse, and additional piping. These costs were factored in as a fraction of the total installed equipment cost. The total indirect costs (TIC), including proratable costs, field expenses, home office and construction, project contingency, and other costs, were calculated based on the TDC. The fixed capital investment (FCI) for the biorefinery is the sum of TDC and TIC. The present design assumes a working capital of 5% of the FCI, and the total capital investment (TCI) is the sum of the FCI and the working capital. The assumptions and costs used to estimate the TDC and TIC were obtained from a previous report [11].

#### Discounted cash flow analysis and minimum jet fuel selling price

The calculated minimum jet fuel selling price assumes a projected net present value of zero at a given fixed annual discount rate (0, 10, 15, or 20%) over a project lifespan of 10 years. For this analysis, the xylitol and formic acid selling prices were fixed at their current market prices. The biorefinery is 60% equity-financed, with 40% being financed from 10-year loans with an annual percentage rate (APR) of 8%. During the start-up period (first year of operation), the biorefinery operates at a 50% reduced capacity. A pretax financial position was assumed due to the complex corporate tax environment, the current favorable depreciation schedules, and the potential for receiving favorable tax exemptions for a new low-carbon industry.

## Conclusion

The present study is the first to present the techno-economic analysis of an integrated biorefinery to produce jet fuel and biobased chemicals from lignocellulosic biomass. We show that co-production of jet fuel, xylitol, and formic acid leads to a jet fuel minimum selling price of $3.13 per gallon, assuming a biorefinery operating at a biomass capacity of 250 ktonne per year assuming a discount rate of 15%. Biomass fractionation, and steam and electricity production, account for a large fraction of the total installed equipment cost. Electricity is an important utility due to the large electricity demand to run the electrochemical reactor used in the production of formic acid, accounting for 59% of the total electricity demand of the biorefinery. Based on our analyses, co-location with a power plant can substantially lower the total capital investment necessary to build the biorefinery and the biorefinery operating costs since the power plant could supply electricity and steam at cost. Finally, sensitivity analysis shows that the selling price of co-products has a major impact on the final jet fuel selling price. In a favorable scenario where xylitol price is 25% higher than its current market price and formic acid is sold at its baseline market price, the minimum jet fuel selling price is $0.64 per gallon, much lower than the DOE target price of $2.50/gallon for SAF by 2030.

## Supplementary Information

**Additional file 1: Figure S1.** Process flow diagram for A100—Biomass fractionation. **Figure S2.** Process flow diagram for A200—Saccharification. **Figure S3.** Process flow diagram for A300—Ethanol production. **Figure S4.** Process flow diagram for A400—Alcohol dehydration, A500 –Oligomerization, and A-600—Hydrogenation. **Figure S5.** Process flow diagram for A900—Boiler and turbogenerator.

## Data Availability

Supporting data to that in the article are provided in the Supplementary Information file.
