# Supplementary material for: Techno-economic analysis of an integrated biorefinery to convert poplar into jet fuel, xylitol, and formic acid
Source: Biotechnol Biofuels Bioprod. 2022 Dec 20;15:143. doi: 10.1186/s13068-022-02246-3 (PMC9768886; doi:10.1186/s13068-022-02246-3)
Supplement: Supplementary file 1 — Additional file 1: Figure S1. Process flow diagram for A100—Biomass fractionation. Figure S2. Process flow diagram for A200—Saccharification. Figure S3. Process flow diagram for A300—Ethanol production. Figure S4. Process flow diagram for A400—Alcohol dehydration, A500 –Oligomerization, and A-600—Hydrogenation. Figure S5. Process flow diagram for A900—Boiler and turbogenerator. [file 13068_2022_2246_MOESM1_ESM.docx]

Additional file 1 Information

Techno-economic analysis of an integrated biorefinery to convert poplar into jet fuel, xylitol, and formic acid

Gabriel V. S. Seufitelli, Hisham El-Husseini, Danielle U. Pascoli, Renata Bura, Richard Gustafson

University of Washington, School of Environmental and Forest Sciences, Seattle, WA, 98195, USA


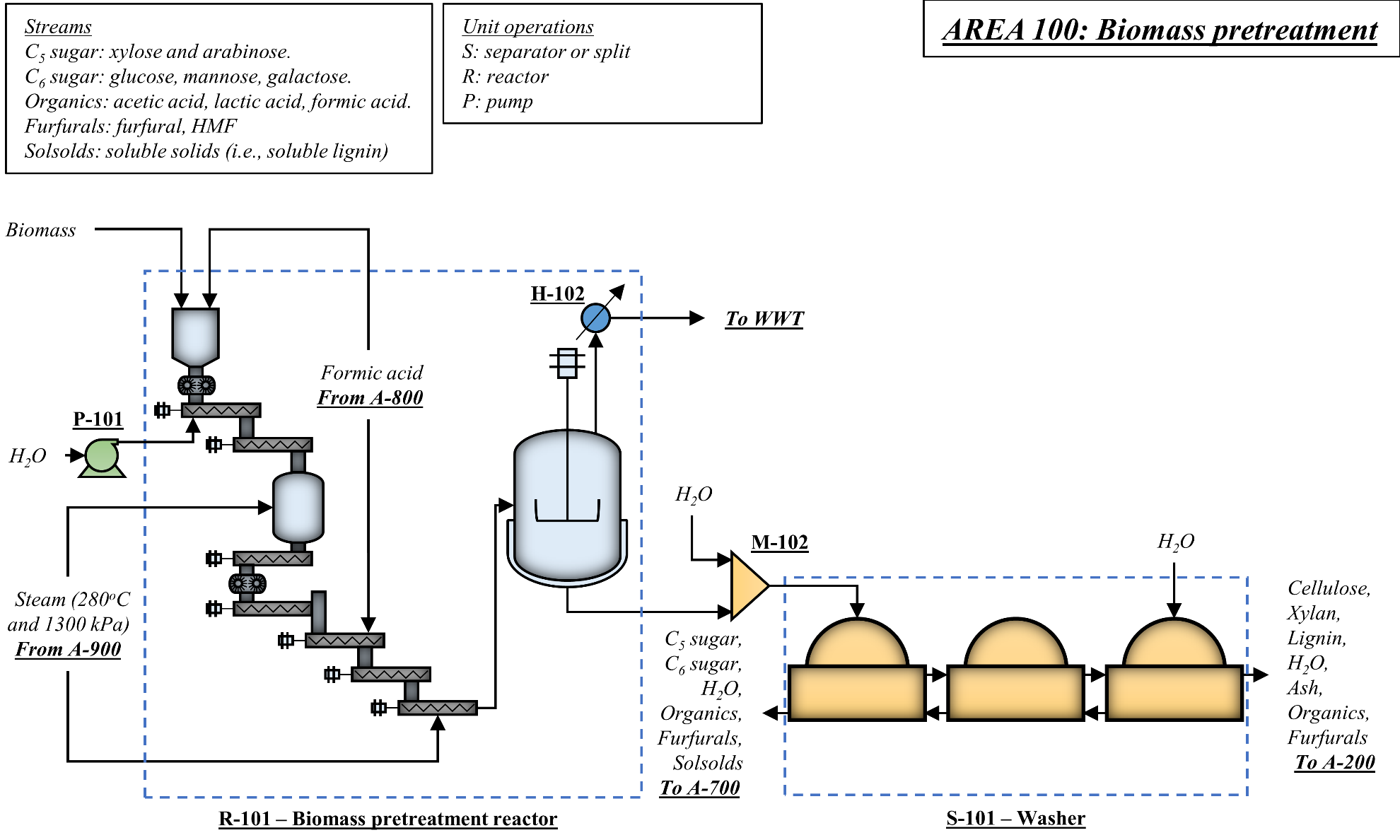


Figure S1: Process flow diagram for A100 – Biomass fractionation.


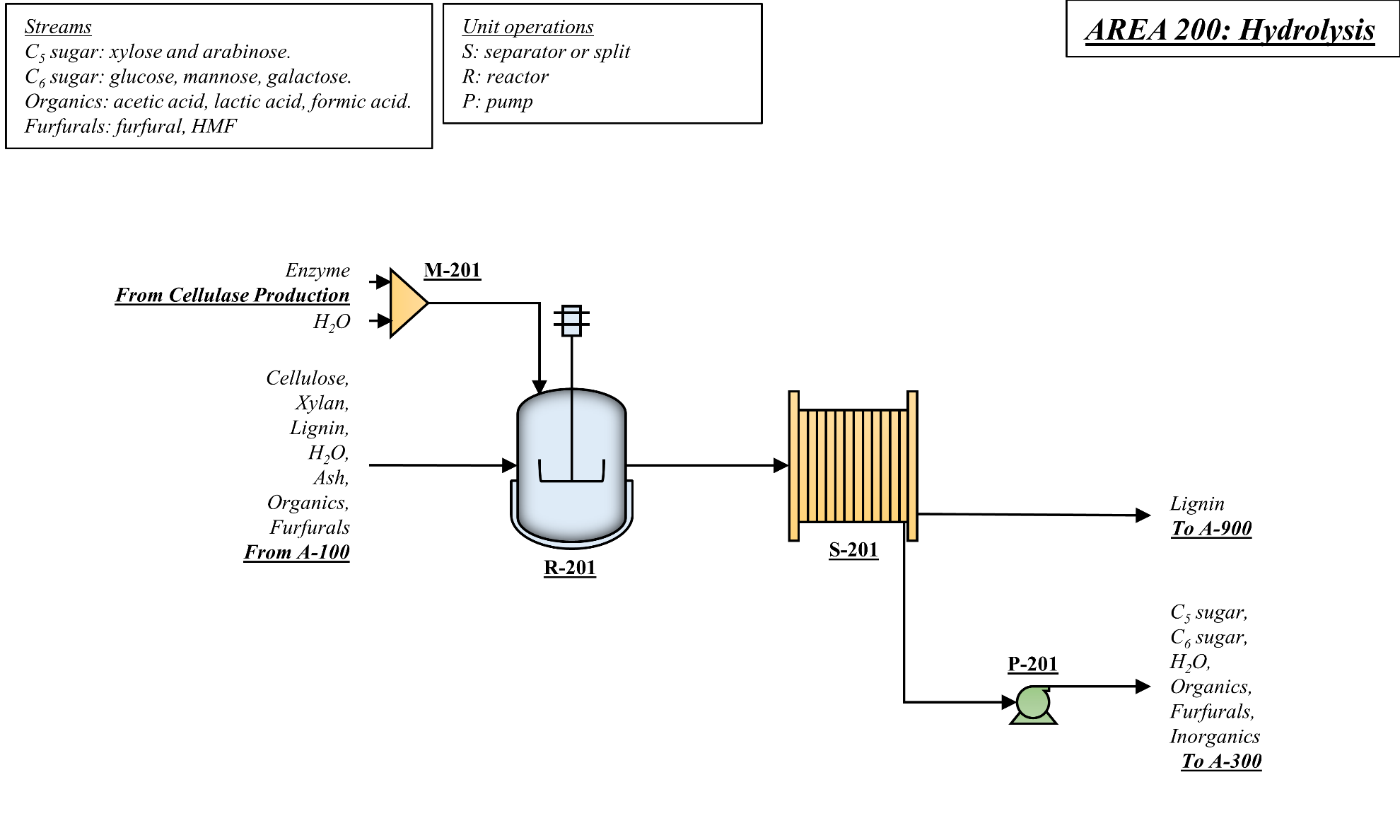


Figure S2: Process flow diagram for A200 – Saccharification.


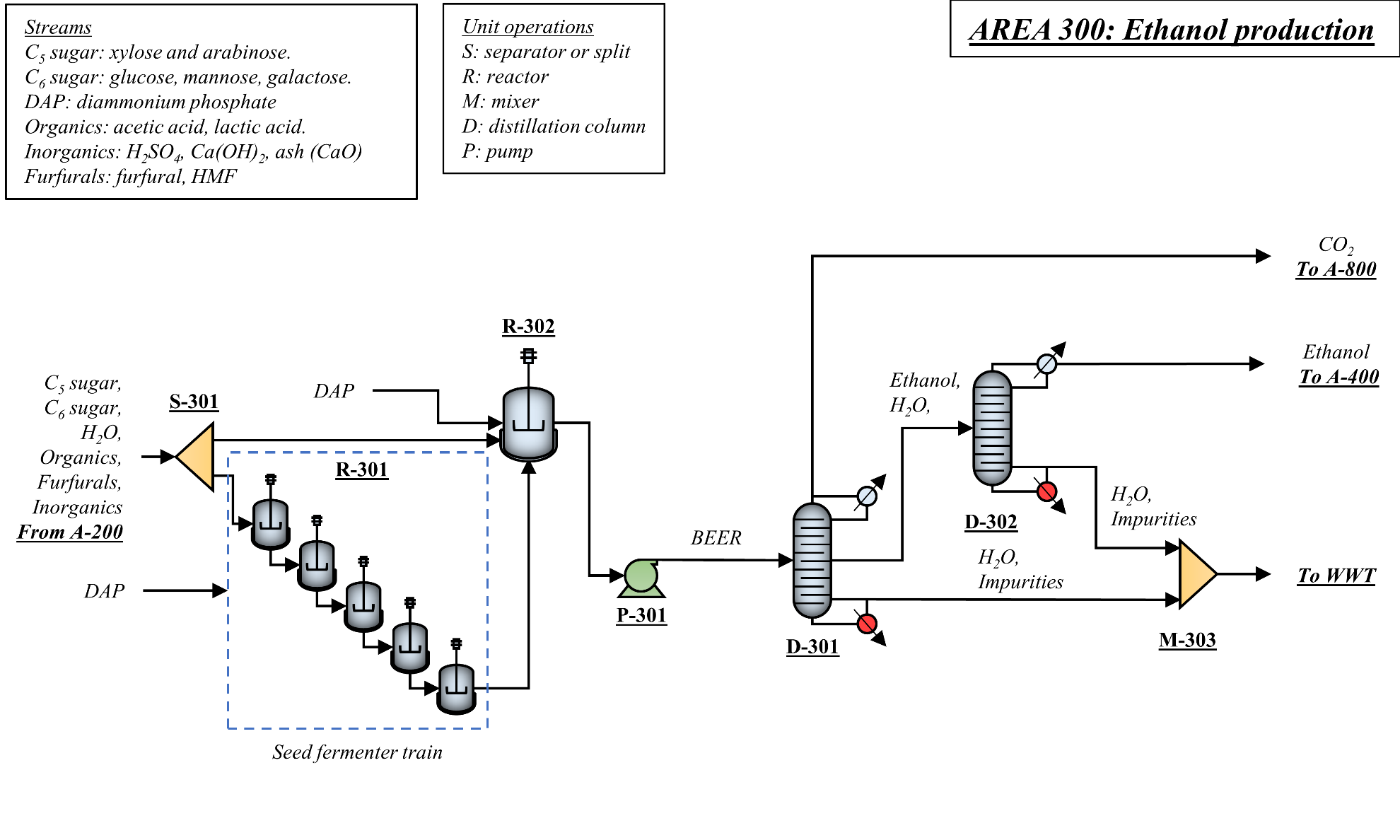


Figure S3: Process flow diagram for A300 – Ethanol production.


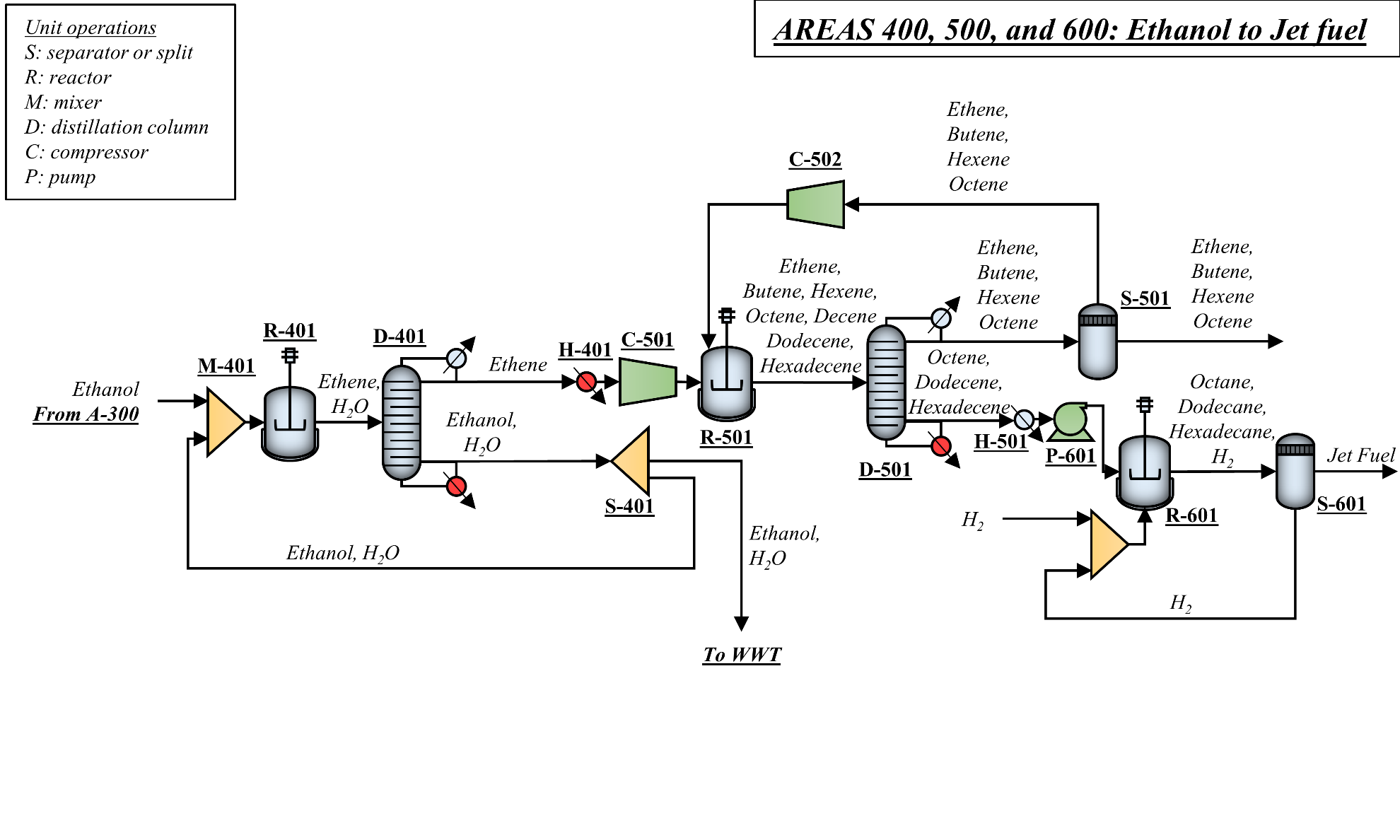


Figure S4: Process flow diagram for A400 – Alcohol dehydration, A500 –Oligomerization, and A-600 – Hydrogenation.


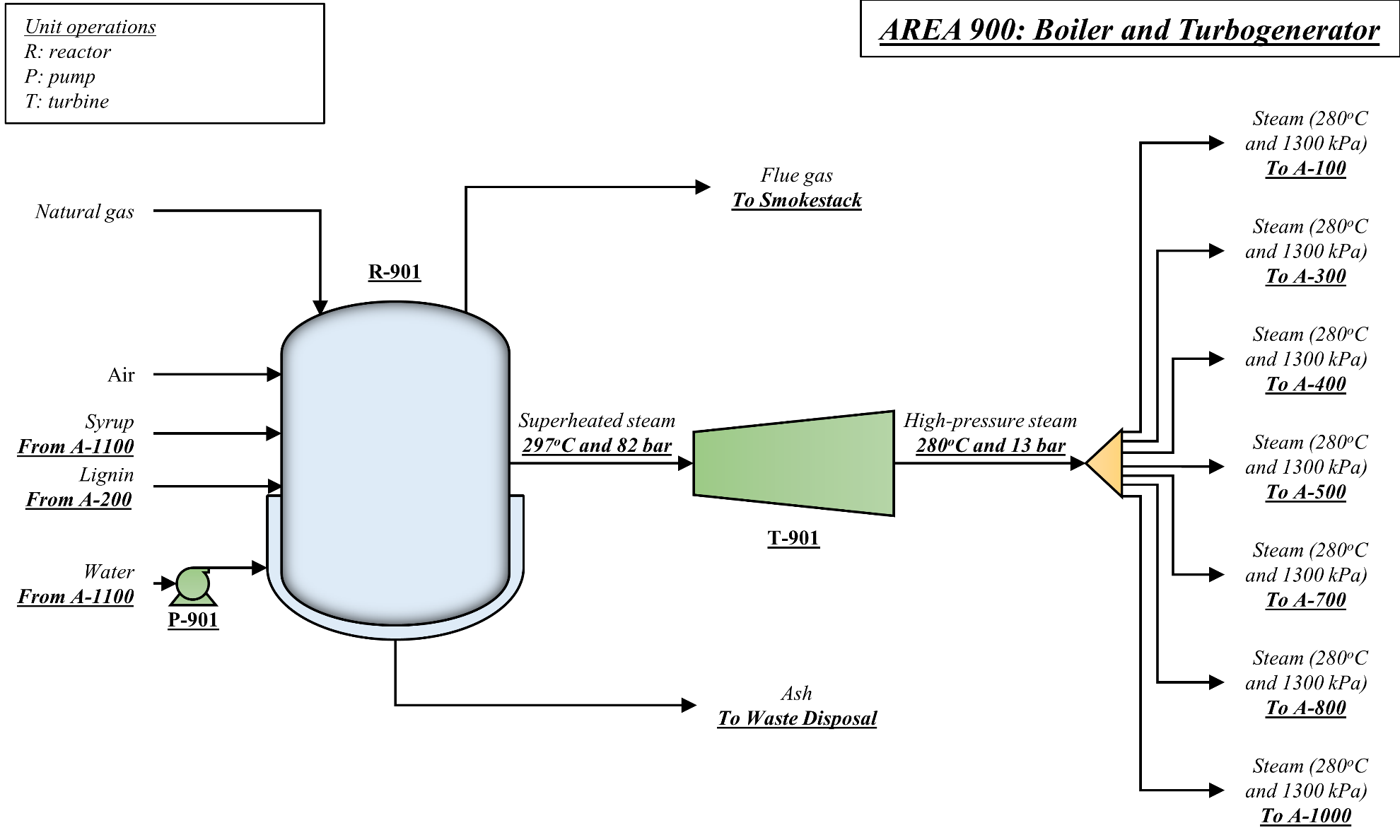


Figure S5: Process flow diagram for A900 – Boiler and turbogenerator.
